# The Trends and Outcomes of Initial Palliative Chemotherapy in Patients with Pancreatic Cancer in Korea Based on National Health Insurance Service Data

**DOI:** 10.3390/jcm13113229

**Published:** 2024-05-30

**Authors:** Dong Kee Jang, Young Ae Kim, Jang Won Lee, Hak-June Kim, Yoon Suk Lee, Jung Won Chun, Jong-Chan Lee, Sang Myung Woo, Jin-Hyeok Hwang

**Affiliations:** 1 Department of Internal Medicine, Seoul National University Boramae Medical Center, Seoul 07061, Republic of Korea; mapmotive@hanmail.net; 2 Division of Cancer Control & Policy, National Cancer Control Institute, National Cancer Center, Goyang 10408, Republic of Korea; elkim7@gmail.com (Y.A.K.); wkwk0123@ncc.re.kr (J.W.L.); multa1lliona@ncc.re.kr (H.-J.K.); 3 Department of Internal Medicine, Inje University Ilsan Paik Hospital, Goyang 10380, Republic of Korea; lys0326@hanmail.net; 4 Research Institute, Center for Liver and Pancreatobiliary Cancer, National Cancer Center, Goyang 10408, Republic of Korea; deli4927@ncc.re.kr; 5 Department of Internal Medicine, Seoul National University College of Medicine, Seoul National University Bundang Hospital, Seongnam 13620, Republic of Korea; ljc0316@naver.com

**Keywords:** pancreatic neoplasms, chemotherapy, survival, big data

## Abstract

**Background/Objectives**: The survival rate of patients with pancreatic cancer (PC) has improved gradually since the introduction of FOLFIRINOX (FFX) and gemcitabine + albumin-bound paclitaxel (GnP) regimens. However, the trends and outcomes of initial palliative chemotherapy before and after the advent of these regimens and their contribution to survival rates are not well understood. This study aimed to investigate this in patients with PC in Korea using claims data from the National Health Insurance Service (NHIS). **Methods:** Patients diagnosed with PC who underwent initial palliative chemotherapy between 2007 and 2019 were identified from the NHIS database. Patient demographics, comorbidities, chemotherapy regimens, and survival rates were analyzed using follow-up data up to 2020. **Results:** In total, 14,760 patients (mean age, 63.78 ± 10.18 years; men, 59.19%) were enrolled. As initial palliative chemotherapy, 3823 patients (25.90%) received gemcitabine alone; 2779 (18.83%) received gemcitabine + erlotinib; 1948 (13.20%) received FFX; and 1767 (11.97%) received GnP. The median survival values were 15.00 months for FFX; 11.04 months for GnP; 8.40 months for gemcitabine alone; and 8.51 months for gemcitabine + erlotinib. The adjusted hazard ratio (aHR) for GnP vs. FFX was 1.291 (95% CI, 1.206–1.383) in the multivariate Cox regression analysis of mortality. Radiation therapy (aHR, 0.667; 95% CI, 0.612–0.728) and second-line chemotherapy (aHR, 0.639; 95% CI, 0.597–0.684) were significantly associated with improved survival. **Conclusions**: Our study found that first-line chemotherapy with FFX was associated with significantly longer survival than the other regimens, although caution is needed in interpreting the results.

## 1. Introduction

The worldwide incidence of pancreatic cancer (PC) in 2020 was approximately 500,000, which was a significant increase (46.7%) from that in 2012. Among the different continents, Asia has the highest incidence and mortality, mainly due to China, which accounts for a large proportion of the cases [1]. As per the Cancer Registration Statistics Program data, there were 247,952 new cancer cases in Korea in 2020. At 8414 cases, PC ranked 8th, accounting for 3.4% of the total cancer incidence. The five-year survival rate for patients with PC in Korea was only 8.6% for those diagnosed between 2006 and 2010. Recently, it has increased to 15.2% for those diagnosed between 2016 and 2020 but remains the lowest among all cancers [2].

The primary reason for the improvement in the survival rate of fatal PC over the recent decade might be the introduction of FOLFIRINOX (FFX) [3] and gemcitabine + albumin-bound paclitaxel (GnP) regimens [4]. Prior to the advent of these two regimens, gemcitabine-based therapy including gemcitabine + erlotinib was the cornerstone of treatment, with unsatisfactory outcomes. Treatments using FFX and GnP have been reimbursed since 2016–2017, one of which was selected for first-line treatment in most patients with PC eligible for palliative chemotherapy in Korea. Additionally, these two regimens can be administered during the first and second alternations. A recent study utilizing the Korean Pancreatic Cancer (K-PAC) registry reported that FFX and GnP showed similar efficacies and toxicities when used as a first-line treatment in patients with metastatic PC. Particularly, patients in the K-PAC registry who received second-line chemotherapy survived for approximately 17 months, highlighting the significance of second-line treatments. Although the analysis of the K-PAC registry yielded actual results for PC treatment in Korea, it does not represent all patients with PC in the country [5].

Although the survival rate for PC is improving, it remains very low, as mentioned previously. Early diagnosis is important to improve the survival rate, but 76% of PC cases are locally advanced or metastatic at the time of diagnosis in Korea [2]. Therefore, choosing the appropriate first-line chemotherapy regimen and following up with second-line chemotherapy is crucial for improving survival. Recently, regimens containing nanoliposomal irinotecan have also become a consideration [6], making it necessary to evaluate the changes in trends and outcomes of first-line chemotherapy from the past to the present.

However, the trends and outcomes of initial palliative chemotherapy before and after the advent of aforementioned regimens and their contributions to survival rates are not well understood. In addition, representative data from all patients with PC in Korea must be analyzed to improve the reliability of the results. This study aimed to investigate the trends and outcomes of initial palliative chemotherapy in patients with PC in Korea using representative claims data from the National Health Insurance Service (NHIS).

## 2. Materials and Methods

### 2.1. Data Source

The database included medical claims data from 2005 to 2020, such as the date of claims, medical record number, diagnosis codes, treatment codes, and prescription information. The NHIS diagnostic codes were derived from the 7th revision of the Korean Standard Classification of Diseases and modified to conform to the 10th revision of the International Classification of Diseases (ICD). The database also included demographic data, such as age, sex, income, and insurance type [7]. All personal data were anonymized for compliance with the Personal Information Protection Act. As the data were anonymized and de-identified for analysis, the requirement for written consent was waived. This study adhered to the ethical principles outlined in the Declaration of Helsinki. All research procedures and ethical considerations were approved by the National Cancer Center Institutional Review Board (No. NCC2021-0091).

### 2.2. Study Population

From the Korean NHIS database from 2005 to 2020, this study included patients who were newly diagnosed with PC between 1 January 2007 and 31 December 2019. The definition of patients with PC was based on ICD-10 code “C25” and claims data containing the rare and intractable diseases cancer registry code (V193) after the diagnosis of PC [8]. The following exclusion criteria were applied: (1) a history of PC diagnosis in 2005–2006 and any cancer diagnosis before the diagnosis of PC; (2) history of pancreatic resection prior to 2006 or before PC diagnosis; (3) missing data; (4) age < 20 years; and (5) history of chemotherapy before insurance approval. All included patients were followed-up from 1 January 2007 to 31 December 2020.

### 2.3. Definitions of Treatment and Outcomes

Chemotherapy regimens were classified into ten groups according to the content of the medical claims data, as follows:

(1) 5-fluorouracil (5-FU) alone: only 5-FU.
(2) 5-FU-based: 5-FU and other anticancer drugs.
(3) TS-1-based: tegafur and uracil or tegafur, gimeracil, and oteracil.
(4) FFX: all three drugs—5-FU, irinotecan, and oxaliplatin.
(5) Gemcitabine alone: only gemcitabine.
(6) Gemcitabine + cisplatin: gemcitabine and cisplatin.
(7) GnP: gemcitabine and nab-paclitaxel.
(8) Gemcitabine + erlotinib: gemcitabine and erlotinib.
(9) Other gemcitabine-based: gemcitabine and other anticancer drugs that were not included in the other groups.
(10) Miscellaneous: if the medical claim did not fit into any of the previous nine groups.

Patients who received a regimen that was different than their initial regimen were defined as having been prescribed “second-line” therapy. Pancreatic resection and radiotherapy were defined using surgical procedure and radiation therapy codes (Supplementary Table S1). We defined initial palliative treatment as any chemotherapy without pancreatic resection. The primary outcome was the survival rate during the follow-up period.

### 2.4. Statistical Analysis

Chi-squared and *t*-tests were used to compare the demographic and clinical characteristics of the study population. Continuous variables were compared using *t*-test, and categorical variables were compared using the chi-squared test. Cox proportional hazards regression models were used to calculate the unadjusted hazard ratios (HRs) and 95% confidence intervals (CIs). For multivariate analysis, age, sex, Charlson Comorbidity Index (CCI), radiation therapy status, and second-line therapy status were included in the model to calculate hazard ratios and 95% CIs. Kaplan–Meier curves and log-rank tests were used to estimate overall survival during the follow-up period. The follow-up period was from the date of PC diagnosis to the date of death or the end of the follow-up period (31 December 2020). *p*-values were calculated using two-sided tests, and *p*-values < 0.05 indicated statistical significance. All statistical analyses were performed using SAS statistical software (version 9.4; SAS Institute, Cary, NC, USA).

## 3. Results

### 3.1. Baseline Characteristics

**Figure 1:**
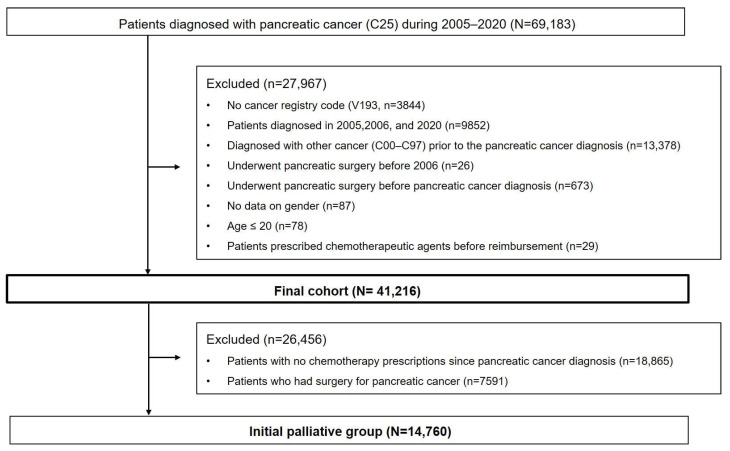
Flowchart of the study population. The process of selecting patients who underwent initial palliative chemotherapy from the database.

**Figure 2:**
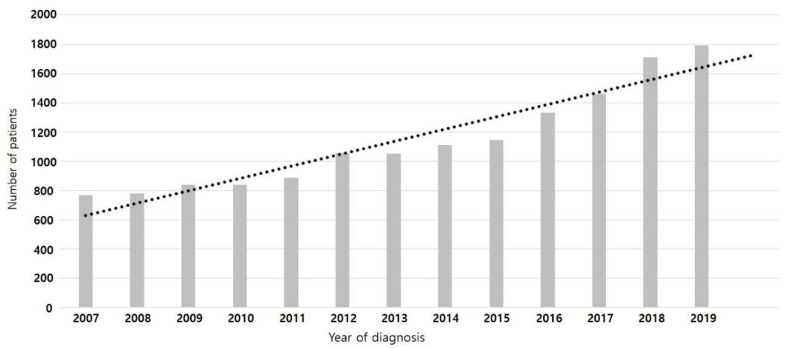
Change in the number of patients who underwent initial palliative chemotherapy for pancreatic cancer by year of diagnosis.

In total, 69,193 patients diagnosed with PC (C25 code) between 2005 and 2020 were identified in the NHIS database. After excluding 27,967 patients, the final PC cohort comprised 41,216 patients. Among them, 14,760 who received initial palliative chemotherapy were included in the analysis (Figure 1). Table 1 shows the baseline characteristics of the patients. The mean age was 63.78 ± 10.18 years, 67.09% (9902/14,760) were aged >60 years, and male patients comprised the majority of the population (N = 8736; 59.19%). Although the percentage of patients with diabetes at the time of PC diagnosis was 28.58%, this figure increased to 66.10% by one year of PC diagnosis. The most common first-line regimen was gemcitabine alone (3823, 25.90%), followed by gemcitabine + erlotinib (2779, 18.83%). FFX (1948, 13.20%) and GnPs (1767, 11.97%) were used in similar proportions. The median follow-up duration was 10.08 months. Figure 2 illustrates the changes in the number of patients who received initial palliative chemotherapy for PC according to the year of diagnosis; this number increased annually.

### 3.2. Survival Outcomes According to First-Line Chemotherapy

**Figure 3:**
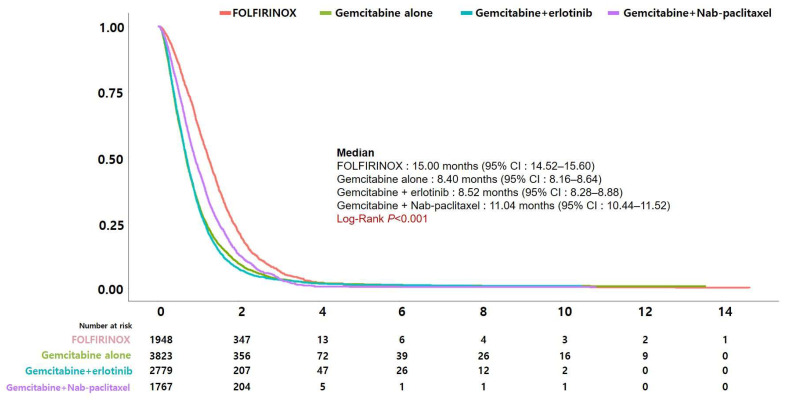
Comparison of survival between patients undergoing chemotherapy with FOLFIRINOX, GnP, gemcitabine alone, and gemcitabine + erlotinib regimens.

**Figure 4:**
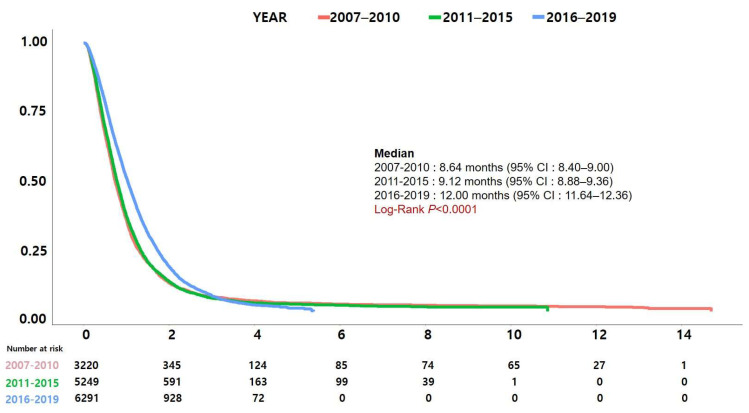
Change in survival by year of diagnosis. The year of diagnosis was divided into three groups (2007–2010, 2011–2015, and 2016–2019) based on the changes in Korea’s major reimbursement policies.

Figure 3 shows Kaplan–Meier curves comparing the survival of patients receiving the four most commonly used regimens (FFX, GnP, gemcitabine alone, and gemcitabine + erlotinib) during the study period. Patients receiving FFX had the longest median survival at 15.00 months, followed by GnP at 11.04 months, gemcitabine + erlotinib at 8.52 months, and gemcitabine alone at 8.40 months. The difference in survival was statistically significant (*p* < 0.001). Survival was significantly different with a log-rank *p* < 0.001 when FFX and GnP were compared separately, but not when gemcitabine alone was compared with gemcitabine + erlotinib (*p* = 0.177). Figure 4 displays the change in survival rates by year of diagnosis. The year of diagnosis was divided into three groups (2007–2010, 2011–2015, and 2016–2019) based on changes in Korea’s major reimbursement policies. Survival rates showed a significant increase over time.

### 3.3. FOLFIRINOX vs. Gemcitabine + Nab-Paclitaxel as First-Line Chemotherapy

Table 2 compares the patients who received FFX (N = 1948) and GnP (N = 1767) regimens. In the FFX group, the patients were significantly younger and a higher proportion of them were receiving radiation therapy (RT) and second-line chemotherapy. However, there were no significant differences in sex or comorbidities, including diabetes. Second-line chemotherapy was administered to 55.75% (1086/1948) and 49.35% (872/1767) of patients in the FFX and GnPs groups, respectively. The most common second-line regimen was gemcitabine alone (14.68%) in the FFX group and TS-1-based regimens (18.11%) in the GnP group. RT was performed more frequently in the FFX group (27.21% vs. 13.30%; *p* < 0.0001).

### 3.4. Factors Associated with Mortality

The results of the univariate and multivariate analyses using Cox proportional hazards regression models to assess the factors associated with mortality are presented in Table 3. In the multivariate analysis, clinical factors, such as increasing age (adjusted HR, 1.010; 95% CI, 1.006–1.014), male sex (adjusted HR, 1.110; 95% CI, 1.036–1.189), and comorbidities, were significantly associated with higher mortality. Among the therapeutic factors, not receiving radiotherapy or second-line chemotherapy was significantly associated with increased mortality. Furthermore, mortality was significantly higher in patients treated with GnP compared to those receiving FFX (adjusted HR, 1.291; 95% CI, 1.206–1.383).

### 3.5. Patients Who Underwent Second-Line Chemotherapy

In the FFX group, 1086 patients (55.75%) received second-line chemotherapy, with 667 (61.42%) of them being ≥60 years and 637 (58.66%) being men. In the GnP group, 872 patients (49.35%) received second-line chemotherapy, with 596 (68.35%) aged ≥60 years and 513 (58.83%) being male. In the FFX group, most patients received gemcitabine alone as second-line therapy, followed by a TS-1-based regimen. Since 2018, the number of patients receiving GnP as second-line therapy increased. In the GnP group, TS-1-based regimens were the most common second-line chemotherapy, followed by FFX (Table 4).

## 4. Discussion

This large-scale cohort study based on claims data included 14,760 patients who underwent initial palliative chemotherapy for PC. To date, this study represents the largest analysis of patients with unresectable PC in Korea. The multivariate analysis revealed that older age, male sex, comorbidities, and not receiving radiotherapy or second-line chemotherapy were associated with increased mortality rates. Additionally, FFX was associated with the best survival outcome compared with other regimens in these patients. Moreover, the survival rate of patients with PC who received initial palliative chemotherapy gradually increased from 2007 to 2019. These findings indicate that the increased use of chemotherapy at diagnosis, particularly FFX and GnP, may contribute to improved survival. Notably, the current study highlights the significant association between second-line chemotherapy and enhanced survival, emphasizing the importance of aggressive chemotherapy in patients with metastatic or unresectable PC.

Here, the FFX group exhibited a median survival of 15.00 months, surpassing the previously recorded survival of 11.1 months [3], whereas the GnP group demonstrated a median survival of 11.04 months, also exceeding the previously recorded survival of 8.5 months [4]. In particular, FFX outperformed GnP significantly, which is consistent with many previous studies [9,10,11,12,13,14,15]. However, some studies did not consider performance status at all [11,12,13], and others had very small numbers of patients included [11,13,15]. Performance status is a factor that must be considered, as it has a significant impact on the selection of initial chemotherapy regimens.

On the other hand, most meta-analyses [16,17,18,19] and some other studies [5,20,21,22,23,24,25,26] have not found a significant difference between the two groups. However, most studies included small numbers of PC patients [20,21,23,24]. The main reason for this discordance is likely the absence of prospective studies thus far; all studies were retrospective, raising the possibility of numerous biases. Furthermore, each study had different inclusion criteria and ethnicities, and, in Korea, the choice of a chemotherapy regimen is heavily influenced by the reimbursement policy. In particular, FFX regimens were covered by insurance for patients with non-metastatic unresectable PC earlier than GnP regimens. A recently published prospective comparison of NALIRIFOX (nanoliposomal irinotecan, oxaliplatin, leucovorin, and fluorouracil) and GnP showed better results in favor of NALIRIFOX [27]; therefore, our results may be considered reliable.

Patients who received second-line chemotherapy consistently had a significantly higher survival rate [21,25,26]. Here, approximately half of the patients in the FFX and GnP groups underwent second-line chemotherapy, potentially contributing to a better survival than that in previous studies. The favorable outcomes in the FFX group could be linked to the reimbursement criteria in Korea, which restricted FFX administration to patients with a good performance status. Furthermore, in Korea, the reimbursement criteria for FFX and GnP initially differed. At that time, FFX was allowed to be administered in both stage 3 and 4 PC cases, whereas GnP was permitted only for stage 4 PC cases.

More than 50% of the patients in our study received second-line chemotherapy, which was associated with a highly significant survival benefit (HR for mortality, 0.639; 95% CI, 0.597–0.684). Patients in the FFX group commonly received gemcitabine alone as a second-line therapy, whereas those in the GnP group were more likely to undergo TS-1-based second-line therapy. The number of patients being administered chemotherapy sequences, such as FFX-GnP or GnP-FFX, increased over time (Table 4). As our database only included patients diagnosed up to 2019, the status of subsequent patients was unknown. However, more patients would adopt this alternate option later. Sequential treatments using FFX and GnP, regardless of the order, showed favorable survival outcomes [5,10]. In contrast, for patients who received gemcitabine-based chemotherapy, including GnP as first-line chemotherapy, second-line treatment with nanoliposomal irinotecan + fluorouracil/leucovorin [6] can be a good option, and recent studies have shown no significant difference compared to that in FFX-based chemotherapy [28,29,30,31]. However, the reimbursement of nanoliposomal irinotecan combination therapy as a second-line treatment in Korea did not begin until 2021; therefore, patients who received this treatment were not included in this study. Therefore, the analysis of databases beyond 2021 may yield better outcomes than the current results.

In the present study, the survival rate of patients who received RT was higher than that of patients who did not receive RT. As patients who underwent surgery or did not receive chemotherapy were not included in our study, RT may mostly have been conducted for palliative purposes. However, considering the nature of the database, with the data of the specific location of RT administration and the purpose of RT not being the latest, it cannot be concluded that RT unequivocally benefits patients receiving palliative chemotherapy. Although RT for PC may be beneficial in cases where it is combined with surgery [32] or performed with stereotactic body radiotherapy [33,34], there is still insufficient evidence to suggest its universal benefit in patients undergoing palliative chemotherapy for PC. However, given the large population of this study, it is likely that RT plays a role in some particular patients with PC receiving palliative chemotherapy. Nonetheless, further research is warranted.

The strength of this study is that it is based on a fully representative database obtained through Korea’s unique NHIS; however, there are obvious limitations owing to the incompleteness of the database itself. Most importantly, the NHIS database contains no information on cancer stage, tumor location, performance status, or adverse events (toxicity). Therefore, although the results of this study showed that FFX was better than any other regimen in terms of survival, it was not possible to compare toxicity, and previous studies have shown various problems due to the toxicity of FFX [5,9,24,26,35]; therefore, it is difficult to conclude that FFX is the best option. Second, because we used an operational definition to identify patients who received initial palliative chemotherapy for PC and to classify the chemotherapy regimen, the inclusion of patients may not have been uniform, and the regimen classification may not have been accurate. In particular, patients classified as receiving second-line treatment with gemcitabine alone in the GnP group may have received gemcitabine alone because of the toxicity of nab-paclitaxel.

## 5. Conclusions

Our study analyzed a large cohort of patients with PC undergoing initial palliative chemotherapy and revealed an increasing trend in the number of patients over time. Additionally, RT and second-line chemotherapy were associated with improved survival rates. Although FFX exhibited the highest survival rates among initial chemotherapy regimens, caution is advised in its interpretation because of the omission of factors such as cancer stage, performance status, and toxicity. Given the recent advances in second-line treatments, including nanoliposomal irinotecan and immunotherapy, future studies should assess their impact on survival.

## Figures and Tables

**Table 1:** Baseline characteristics (N = 14,760).

Variable	Value
Age (y)	63.78 ± 10.18
Male sex, n (%)	8736 (59.19)
Diabetes at pancreatic cancer diagnosis, n (%)	4219 (28.58)
Diabetes within 1 year of pancreatic cancer diagnosis, n (%)	9756 (66.10)
Charlson Comorbidity Index, n (%)	
0–2	8201 (55.56)
3–6	6004 (40.68)
7-	555 (3.76)
First-line chemotherapy regimen, n (%)	
Gemcitabine alone	3823 (25.90)
Gemcitabine + erlotinib	2779 (18.83)
5-FU alone	462 (3.13)
5-FU-based	914 (6.19)
TS-1-based	466 (3.16)
Gemcitabine + nab-paclitaxel	1767 (11.97)
FOLFIRINOX	1948 (13.20)
Gemcitabine + cisplatin	674 (4.57)
Other gemcitabine-based regimens	1005 (6.81)
Miscellaneous	922 (6.25)
Second-line chemotherapy, n (%)	7875 (53.35)
Follow-up duration (month), median (IQR)	10.08 (5.52, 16.92)

**Table 2:** Comparison of patients treated with FOLFIRINOX and gemcitabine + nab-paclitaxel.

	FOLFIRINOX (N = 1948)		GnP (N = 1767)		*p*-Value
	N	%	N	%	
Total					
Age					
Mean ± SD	62.57 ± 9.59		64.89 ± 9.61		<0.0001
Median (Q1, Q3)	63 (57, 70)		65 (59, 72)		
<60	697	35.78	482	27.28	<0.0001
≥60	1251	64.22	1285	72.72	
Gender					
Male	1147	58.88	1033	58.46	0.7950
Female	801	41.12	734	41.54	
Diabetes					
Yes	1300	66.74	1197	67.74	0.5139
No	648	33.26	570	32.26	
CCI					
0 ≤ CCI < 3	1121	57.55	958	54.22	0.1186
3 ≤ CCI < 7	755	38.76	742	41.99	
CCI ≥ 7	72	3.70	67	3.79	
Radiation therapy					
Yes	530	27.21	235	13.30	<0.0001
No	1418	72.79	1532	86.70	
Second-line chemotherapy					
Yes	1086	55.75	872	49.35	<0.0001
No	862	44.25	895	50.65	
Gemcitabine alone	286	14.68	291	16.47	
Gemcitabine + erlotinib	144	7.39	9	0.51	
5-FU alone	154	7.91	103	5.83	
5-FU-based	162	8.32	56	3.17	
TS-1-based	181	9.29	320	18.11	
GnP	61	3.13	0	0.00	
FOLFIRINOX	0	0.00	32	1.81	
Gemcitabine + cisplatin	40	2.05	3	0.17	
Other gemcitabine-based regimens	7	0.36	43	2.43	
Miscellaneous	51	2.62	15	0.85	

GnP, gemcitabine + nab-paclitaxel; CCI, Charlson Comorbidity Index.

**Table 3:** Factors associated with mortality.

Variables	Univariate		Multivariate	
	HR	95% CI	aHR	95% CI
Age	1.015	1.001–1.019	1.010	1.006–1.014
Sex				
Female	Ref.		Ref.	
Male	1.103	1.030–1.181	1.110	1.036–1.189
CCI				
0 ≤ CCI < 3	Ref.		Ref.	
3 ≤ CCI < 7	1.124	1.049–1.205	1.051	0.979–1.128
CCI ≥ 7	1.408	1.182–1.677	1.284	1.076–1.531
Radiation therapy				
No	Ref.		Ref.	
Yes	0.592	0.544–0.645	0.667	0.612–0.728
Second-line therapy				
No	Ref.		Ref.	
Yes	0.610	0.570–0.653	0.639	0.597–0.684
First-line therapy				
FOLFIRINOX	Ref.		Ref.	
GnP	1.400	1.308–1.497	1.291	1.206–1.383

HR, hazard ratio; CI, confidence interval; aHR, adjusted hazard ratio; CCI, Charlson Comorbidity Index; GnP, gemcitabine + nab-paclitaxel.

**Table 4:** Second-line chemotherapy by year in the FOLFIRINOX and gemcitabine + nab-paclitaxel groups.

	FFX (N = 1086)									
Diagnosis Year	Gemcitabine Alone	Gemcitabine + Erlotinib	5-FU Alone	5-FU-Based	TS-1-Based	GnP	FFX	Gemcitabine + Cisplatin	Other Gemcitabine-Based Regimens	Miscellaneous
2007	1	0	1	0	0	0	0	0	0	0
2008	0	0	0	0	0	0	0	0	0	0
2009	0	0	0	0	1	0	0	0	0	0
2010	0	0	0	0	0	0	0	0	0	0
2011	0	0	0	0	0	0	0	0	0	0
2012	1	0	0	0	0	0	0	0	0	0
2013	0	0	0	0	0	0	0	0	0	0
2014	0	0	0	0	1	0	0	0	0	0
2015	1	0	0	1	1	0	0	0	0	0
2016	4	6	0	5	6	0	0	2	0	0
2017	62	41	42	40	48	8	0	9	4	10
2018	68	50	50	47	65	20	0	22	2	17
2019	149	47	61	69	59	33	0	7	1	24
Total	286	144	154	162	181	61	0	40	7	51
	GnP (N = 872)									
2007	0	0	0	0	0	0	0	0	0	0
2008	0	0	0	0	0	0	0	0	0	0
2009	0	0	0	0	0	0	0	0	0	0
2010	0	0	0	0	0	0	0	0	0	0
2011	0	0	0	0	1	0	0	0	0	0
2012	0	0	0	0	0	0	0	0	0	0
2013	0	0	0	0	0	0	0	0	0	0
2014	0	0	0	0	0	0	0	0	0	0
2015	4	0	0	0	0	0	0	0	4	0
2016	36	0	0	1	2	0	1	0	36	1
2017	2	1	18	11	49	0	46	0	2	2
2018	0	7	46	27	158	0	140	2	0	8
2019	1	1	39	17	110	0	104	1	1	4
Total	43	9	103	56	320	0	291	3	43	15

FFX, FOLFIRINOX; GnP, gemcitabine + nab-paclitaxel.

## Data Availability

The data analyzed in this study are only accessible in restricted places, making them difficult to share, even if requested.

## Supplementary Materials

The following supporting information can be downloaded at: https://www.mdpi.com/article/10.3390/jcm13113229/s1, Table S1. Surgical procedures and radiotherapy codes.
