# Supplementary material for: The Trends and Outcomes of Initial Palliative Chemotherapy in Patients with Pancreatic Cancer in Korea Based on National Health Insurance Service Data
Source: J Clin Med. 2024 May 30;13(11):3229. doi: 10.3390/jcm13113229 (PMC11172641; doi:10.3390/jcm13113229)
Supplement: Supplementary file 1 [file jcm-13-03229-s001.zip › jcm-3000239-supplementary.pdf]

Supplementary Table S1. Surgical procedures and radiotherapy codes

Type	Name	Procedure codes
Surgery	Total pancreatectomy	Q7561
	Isolated head pancreatectomy	Q7562
	Subtotal pancreatectomy	Q7563
	Segmental resection	Q7564
	Distal pancreatectomy	Q7565
	Wedge resection	Q7566
	Spleen preserving distal pancreatectomy	Q7567
	Pancreaticoduodenectomy, Whipple's	Q7571
	Pyloric preserving pancreaticoduodenectomy	Q7572
Radiation	Computerized radiotherapy planning: paralled opposed ports	HD014
	Computerized radiotherapy planning: non-paralled opposed ports, more than 3 ports	HD015
	Computerized radiotherapy planning: 3-dimensional conformal radiation therapy	HD018
	Computerized radiotherapy planning: stereotactic radiosurgery and radiotherapy	HD019
	Computerized radiotherapy planning: proton therapy planning	HD020
	Design and construction of therapeutic devices: shielding Block	HD031
	Design and construction of therapeutic devices: Compensator or Bolus	HD032
	Design and construction of therapeutic Devices: immobilization devices	HD033
	Computerized radiotherapy planning: intensity-modulated radiation therapy planning	HD041
	Teletherapy: low-energyparalled opposed ports	HD054
	Teletherapy: middle-energy-paralled opposed ports	HD055
	Teletherapy: high-energy-paralled opposed ports	HD056
	3-dimensional conformal therapy	HD061
	Fractionated stereotactic radiotherapy	HD110
	Body stereotactic radiosurgery: linac	HD111
	Body stereotactic radiosurgery: linac	HD112
	Proton therapy	HD121
	Computerized radiotherapy planning: paralled opposed ports	HD414
	Computerized radiotherapy planning: non-paralled opposed ports, more than 3 ports	HD415

	Computerized radiotherapy planning: 3-dimensional conformal radiation therapy	HD418
	Computerized radiotherapy planning: stereotactic radiosurgery and radiotherapy	HD419
	Computerized radiotherapy planning: proton therapy planning	HD420
	Computerized radiotherapy planning: intensity-modulated radiation therapy planning	HD441
	Intensity-modulated radiation therapy	HZ271
